# Contribution of Infrapatellar Fat Pad and Synovial Membrane to Knee Osteoarthritis Pain

**DOI:** 10.1155/2019/6390182

**Published:** 2019-03-31

**Authors:** Elisa Belluzzi, Elena Stocco, Assunta Pozzuoli, Marnie Granzotto, Andrea Porzionato, Roberto Vettor, Raffaele De Caro, Pietro Ruggieri, Roberta Ramonda, Marco Rossato, Marta Favero, Veronica Macchi

**Affiliations:** ^1^Musculoskeletal Pathology and Oncology Laboratory, Department of Orthopaedics and Orthopaedic Oncology, University of Padova, Padova, Italy; ^2^Institute of Human Anatomy, Department of Neuroscience, University of Padova, Italy; ^3^L.i.f.e.L.a.b. Program, Consorzio per la Ricerca Sanitaria (CORIS), Veneto Region, Via N. Giustiniani 2, 35128 Padova, Italy; ^4^Clinica Medica 3, Department of Medicine, DIMED, University of Padova, Italy; ^5^Department of Orthopaedics and Orthopaedic Oncology, University of Padova, Padova, Italy; ^6^Rheumatology Unit, Department of Medicine, DIMED, University Hospital of Padova, Italy

## Abstract

Osteoarthritis (OA) is the most common form of joint disease and a major cause of pain and disability in the adult population. Interestingly, there are patients with symptomatic OA displaying pain, while patients with asymptomatic OA that do not experience pain but show radiographic signs of joint damage. Pain is a complex experience integrating sensory, affective, and cognitive processes related to several peripheral and central nociceptive factors besides inflammation. During the last years, the role of infrapatellar fat pad (IFP), other than the synovial membrane, has been investigated as a potential source of pain in OA. Interestingly, new findings suggest that IFP and synovial membrane might act as a functional unit in OA pathogenesis and pain. The present review discuss the role of IFP and synovial membrane in the development of OA, with a particular focus on pain onset and the possible involved mediators that may play a role in OA pathology and pain mechanisms. Inflammation of IFP and synovial membrane may drive peripheral and central sensitization in KOA. Since sensitization is associated with pain severity in knee OA and may potentially contribute to the transition from acute to chronic, persistent pain in knee OA, preventing sensitization would be a potentially effective and novel means of preventing worsening of pain in knee OA.

## 1. Introduction

Osteoarthritis (OA) is the most common form of chronic joint disease and a major cause of pain and disability (typical clinical OA features) in the adult population, affecting females more than males and disabling nearly 27 million of adults in USA [1, 2]. Anatomically, the main target-joint is the knee with a global prevalence of 3.8% [2]. OA was ranked as the 11^th^ highest contributor to global disability and 38^th^ highest in disability-adjusted life year in 2010 [3]. According to the World Health Organization, worldwide 9.6% of men and 18.0% of women aged over 60 years have symptomatic OA [4]. Moreover, being a progressive condition, it leads to functional decline and loss of quality of life, with important and huge impact on healthcare and social costs [5]. OA also determines a significantly high economic burden due to the increase of both direct (healthcare visits and total joint replacements) and indirect costs (productivity losses and cares) [6]. To date, there are no disease-modifying drugs for this pathology and the available treatments are aimed at pain relief, being the surgical joint replacement the only option for the end-stage disease. From a physiopathological perspective, knee OA (KO) is now considered as a whole joint disease that involves not only cartilage but also the meniscus [7], the subchondral bone [8], the synovial membrane [9], and the infrapatellar fat pad (IFP) that are stricken [10].

The typical disease-related features include loss of articular cartilage, subchondral bone sclerosis, osteophytes, and low-grade synovitis [11]. However, its etiopathogenesis has not been fully clarified so far [11]. Several local and systemic risks factors are recognized to have an active role in the development of the pathology [12].

**Figure 1:**
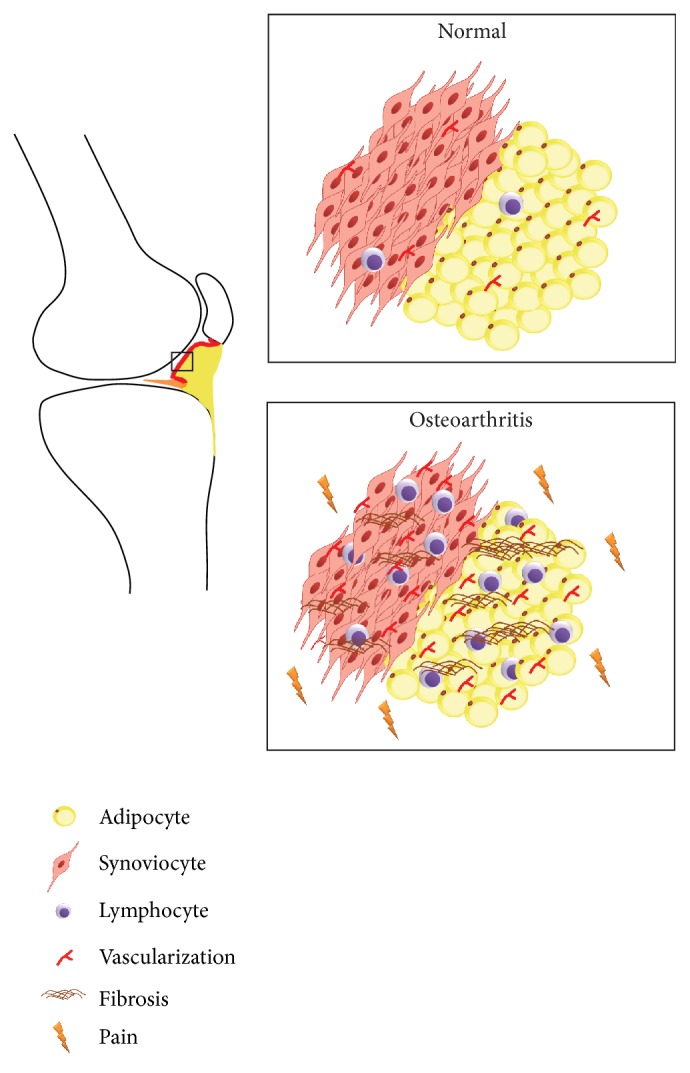
*Infrapatellar fat pad and synovial membrane in healthy and osteoarthritic condition*. In osteoarthritis there is an increase of fibrosis, lymphocytic infiltration, and vascularization compared to normal tissues. These changes contribute to OA pain.

OA can be symptomatic or asymptomatic. In particular, people with symptomatic OA experience pain causing disability, while people with asymptomatic OA display radiographically structural joint damage without pain [13]. To date, the explanations as well as the factors differentiating symptomatic from asymptomatic OA still remain elusive [13]. Typically, pain is the reason why patients ask for a medical consultation [14]. However, unfortunately, most of the patients continue to present pain even after conventional drug treatments and, in some cases, also after total joint replacement [14]. Therefore, there is an urgent and pressing need to unravel not only the etiopathogenesis but also the pathways leading to OA pain in order to identify specific targets useful to develop new effective treatments for its management. In this context, careful attention should be paid to a new emerging concept reconsidering the relation between the IFP and the synovial membrane, which are tissues innervated and well vascularized [15, 16]. Recently, it has also been shown that IFP in OA represents a source of inflammatory molecules and is more inflamed and vascularized compared to nonosteoarthritic controls (Figure 1) [17–19]. Moreover, it has been known that IFP can be the cause of anterior knee pain [20]. OA synovial membrane is characterized by increased hyperplasia, fibrosis, vascularization, and immune cells infiltration as well as inflammatory molecules and neuropeptides production (Figure 1) [21]. Synovial inflammation correlates with OA symptoms and is characterized by increased responsiveness of peripheral nociceptive neurons contributing to pain sensitivity [21, 22].

According to Bastiaansen-Jenniskens et al., IFP may contribute to the development of synovial fibrosis in the knee joint promoting cell migration and proliferation [23]. In addition, recent evidence suggests that IFP and synovial membrane may be considered as the constitutive elements of a single anatomofunctional unit and not two separate tissues [15, 16]. To this regard, our group has recently discussed the basis of such interplay considering macroscopic anatomy, imaging, and histopathology up to molecular biology evidence [24]. Thus, basing on this assumption, the aim of this review is to discuss the role of IFP and synovial membrane in the development of OA, with a particular focus on pain onset and the possible involved mediators.

## 2. Methods

### 2.1. Narrative Review Search Strategy

The review search has been conducted in order to describe the involvement of the functional unit IFP-synovial membrane to the onset of knee pain mechanisms; no limits in terms of time or language were adopted. Moreover, both reviews and original papers that evaluated the relationship between IFP, synovial membrane, and pain in OA were considered. In October 2018, PubMed and Scopus were queried selecting the terms knee, osteoarthritis (OR osteoarthritis features), pain, sensitivity, infrapatellar fat pad, synovial membrane, magnetic resonance imaging, and pain mediators as searching terms. In particular, multiples unrestricted “free-text” searches were performed combining the terms (osteoarthritis AND knee pain sensitivity), (infrapatellar fat pad AND osteoarthritis), (synovial membrane AND knee osteoarthritis), (osteoarthritis features AND magnetic resonance imaging), and (osteoarthritis AND pain mediators). Other reviews and original articles were also obtained consulting the references of the previously articles which were selected from the databases. In parallel, we have also referred to our experience on the theme as well as to both published and unpublished data of our research group in this topic. Duplicates as well as nonrelevant records were removed. Moreover, significant publications were selected among the references of the previous articles.

## 3. The Role of the IFP-Synovial Membrane as a Functional Unit in the Pathogenesis of OA

### 3.1. Anatomy and Function of the IFP

**Figure 2:**
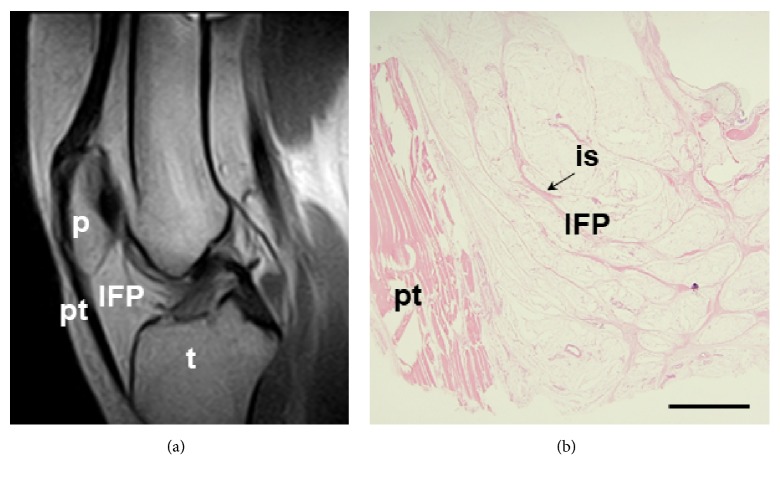
*Anatomy of the infrapatellar fat pad (IFP).* (a) Sagittal section of magnetic resonance, showing the IFP and its location with respect to patella (p), patellar tendon (pt), and tibia (t). (b) Microscopic sagittal image of the IFP, showing the organization in small lobuli and thin interlobular septa (is, black arrow) by Hematoxylin and Eosin staining. Scale bar = 1,200 *μ*m.

Within the knee joint, specifically between the joint capsule and the synovial membrane, several fat pads can be recognized. These are the quadriceps fat pad and the prefemoral fat pad that constitute the suprapatellar fat pad, the posterior fat pad, and the IFP [24, 25]. Among fat pads, IFP, which is also known as Hoffa's fat pad, is the one that has received a particular attention in recent years (Figure 2) [26]. Anatomically, it is formed by a central body, showing the presence of two extensions (i.e., medial and lateral) along with the superior tag. In its inferior part, the ligamentum mucosum is recognizable along with two clefts that are sometimes identifiable, opening posteriorly (i.e., the horizontal one) and superiorly (i.e., the vertical one) below the patella [24, 25].

The IFP is located between the patellar tendon, the intercondylar notch, the inferior pole of the patella, the anterior tibial plateaus, the anterior horns of both menisci, and the anterior surface of the femur [20, 27]. Regarding its posterior aspect, it is lined by the synovial membrane [24]. From the microscopic point of view, the IFP histological characterization shows the presence of lobules (mean diameter of 1.15 ± 0.11 mm) delineated by thin connective septa (0.22 ± 0.034 mm) consistent with the typical structural features of white adipose tissue [28]. Adipocytes make up most of the cell population; but also fibroblasts, which are responsible for the production of the extracellular matrix and immune cells (i.e., macrophages, mast cells, and lymphocytes) are recognizable and exert an important active role in the development of OA [18].

As concerns blood supply, the IFP has numerous vessels forming a framework with the fibrous tissue [27]. In particular, two vertical arteries, located posterior to the lateral edges of the patellar tendon are recognizable, being themselves interconnected by three horizontal arteries and supplied by the superior and inferior genicular arteries. An additional anastomotic artery may be present within the infrapatellar synovial fold connecting the medial genicular artery and the middle or superior horizontal artery [29]. While the peripheral area of the pad is well supplied, there is paucity of vascularity in the central zone [30].

Interestingly, the rich vascular supply found in the IFP validates the hypothesis that the IFP can aid with healing the anterior cruciate ligament and other nearby structures, but it also supports the model of fibrosis and subsequent knee pain or stiffening after IFP injury [31].

Many studies demonstrate that IFP is highly innervated by the posterior articular branch of the tibial nerve [18]. In particular, Gardner describes branches for IFP rising from the saphenous, tibial, and obturator nerves, and from the nerve for the vastus medialis, addressed to the anteromedial portion of the IFP. The anterolateral part of the pad is innervated by articular branches from the nerve for the vastus lateralis, the tibial nerve, recurrent peroneal nerve, and common peroneal nerve. These branches accompany blood vessels throughout the IFP [18]. Some of these multiple fine fibers terminate by innervating its synovial lining [32]. Because of its rich innervation and relationship with the highly innervated synovial membrane, the IFP may be considered a potent source of pain [32].

To date, the actual function of the IFP in the knee is not fully elucidated, representing an interesting research field [33]. In addition it was considered as a mere deformable space filler for a long time, acting like a shock absorber during movement of the knee joint and a bystander in KOA, to support patella tendon blood supply [18, 30, 34]. This idea was supported by the important presence of constitutive collagen stroma and mainly by its position [35]. IFP cushioning role in the knee joint facilitates distribution of synovial fluid and dampens mechanical stresses during articular activity. Interestingly, its response under stress conditions is related to its anatomical localization, with a peculiar mechanical behavior different from that shown by the subcutaneous adipose tissue of the abdomen, as recently confirmed also by our group [33]. Moreover, we recently showed a decrease of IFP volume, depth, femoral, and tibial arch lengths in moderate and end-stage OA compared to controls as well as a difference in IFP hypointense signal [36]. Interestingly, no difference was found in suprapatellar fat pad between the groups, supporting the specific role of IFP in OA [36].

Awareness about IFP role is extremely important also in clinical practice. In fact, IFP preservation or resection during TKA is still a matter of debate and no definitive guidelines are available [37]. Even though IFP resection during knee surgery allows easy access and visualization, evidence suggests that its preservation is advantageous [38]. In a study by Tanaka et al., the authors compared the clinical results of IFP resection and synovectomy in patients undergoing TKA for rheumatoid arthritis [39]. The group of patients experiencing synovectomy including IFP resection showed an increased incidence of anterior knee pain with a significant limitation of knee motion, slight quadriceps weakness, and significant shortening of patellar tendon length in comparison with patients without infrapatellar synovectomy or IFP resection [39]. Later, Lemon et al. evaluated the length of the patellar tendon in patients experiencing or not the resection of the IFP in case of TKA [40]. According to the radiographic data, the patella tendon was significantly shortened in the IFP-resected group with no change in the IFP-preserved group at three years postoperatively [40]. Hence, even though the patella tendon length does not always shorten after TKA, IFP preservation may be a protective factor in preventing such occurrence. Moverley et al. suggested that excision of the IFP during TKA surgery should be avoided unless absolutely necessary because of its important contribution in the biomechanical equilibrium of the knee [38]. More recently, a systematic review reported that IFP preservation leads to a reduction in long-term knee pain [41]. However, there is a need of level one randomized controlled trials, which would shed light on this issue [41, 42].

Next to a mechanical role, IFP preservation under extreme starvation conditions points out its implication in knee joint homeostasis [43], in particular, in mediating joint inflammation [10, 30, 44].

Several studies have suggested a pivotal role of the IFP in the development of OA [5]. The mediators involved in the inflammatory process of the joints also determine the onset of structural changes of the IFP, which is characterized by an increase of inflammatory infiltration, vascularization, and thickness of the interlobular septa as well documented at both cellular and histological levels in OA [25]. In particular, in the OA IFP, higher vascular endothelial growth factor (VEGF) levels were associated with an increased number of vessels in the OA IFP while higher monocyte chemotactic protein 1 (MCP-1) and IL-6 protein levels were related to higher grades of inflammatory infiltration. In parallel, a consistent loss of adipocytes, extensive synovial proliferation, and subsynovial fibrosis were also assessed [25].

### 3.2. Anatomy and Function of the Synovial Membrane

The spaces of diarthrodial joints, bursae, and tendon are covered by a specialized mesenchymal tissue, that is, the synovial membrane. From the microscopic point of view, this membrane has two layers: (a) the intimal and (b) subintimal layer. Typically, in healthy subjects, the cross-sections of the intimal and subintimal layers show 20-40 mm and up to 5 mm thickness, respectively. However, at many sites there is no discrete membrane, especially where subintima consists of fibrous tissue or adipose tissue [27].

The intima inner layer is characterized by the presence of one or two sheets of macrophages (type A synoviocytes) or fibroblast-like synoviocytes (type B synoviocytes). Type A synoviocytes are recognizable in the upper part of synovial lining, showing a surface rich in microvilli and microplicae. They are positive for CD163 and CD68 but not for CD14^+/lo^ with a nonspecific esterase activity; typically, they proliferate in inflammatory conditions. Differently from type A synoviocytes, type B synoviocytes express both the surface marker CD55 and class II major histocompatibility molecules, proving a key role in early phases of immune responses in the synovial membrane. Type B synoviocytes are found further from the synovial lining; they produce mainly hyaluronic acid (one of the main components of cartilage ECM), which binds to the cell receptor CD44. Moreover, they are active in the production of lubricin having role in articular cartilage surface-protection.

The subintima outer layer shows two to three layers of synoviocytes lying over loose connective tissue rich in fibroblasts, secreting collagen, and other proteins of the ECM. It has few macrophages and lymphocytes, fat cells, and blood vessels, which are active in providing nutrients to both the synovial membrane and the cartilage tissue [45, 46]. Under pathological conditions, synovial macrophages may contribute to cartilage destruction due to prolonged production of proinflammatory cytokines or through the formation of osteophytes by the release of the transforming growth factor-beta (TGF-*β*)3 and bone morphogenetic protein- (BMP-) 2 and BMP-4 [45].

The synovium has a rich microcirculation which allows the transfer of small and large molecules to and from the blood into synovial tissues [47]. While capillaries occur just below or within the intima, small venules are prominent within the normal synovium. Still deeper in the subintimal layer there are larger venules together with arterioles and lymphatics, forming an anastomosing array. Capillaries and postcapillary venules average a density of 240/mm^2^ [48]. In accordance with what occurs in many other sites, enlargement of endothelial cells as well as microvascular proliferation has been observed during inflammation. To date, no specific vascular markers for the inflamed synovial membrane have been found; however, it has been hypothesized the existence of specialized lymphocyte trafficking pathways chemokine-receptor-based [46].

As demonstrated by Xu et al., both normal and arthritic synovial tissues show lymphatic vessels which are more represented where there is both edema and an increase in inflammatory cells in the subintima [47].

The normal synovial membrane has a rich nerve supply and, including the sympathetic nervous system, most of the nerves are recognizable in proximity of the vascular networks despite not extending deep into the intimal layers [46].

The role of the synovial membrane is quite clear, being dedicated to the promotion of skeletal movement by the production of the synovial fluid (SF). Cells from the synovial membrane intimal layer secrete the SF, which facilitates low-friction and low-wear articulation filling the synovial cavity, lubricating cartilage, and tendon surfaces [24] and sustain chondrocyte activity and nutrition [45].

It is noteworthy that OA is often associated with synovial inflammation [21]; this feature is mainly represented in the proximity of pathologically damaged cartilage and bone, being responsible for disease progression and symptoms (i.e., joint swelling, pain, and effusion) [21]. In addition to its involvement in early stages of the disease, synovial inflammation is also thought to be secondary to penetration of osteocartilaginous fragments and inflammatory/catabolic factors in the synovial cavity [21]. Interestingly, all OA joint tissues are able to stimulate synoviocytes and might be involved in the progression of OA synovitis [17].

According to the histological characterization of the tissue, the hallmarks of the synovial membrane in case of OA are hypertrophy and hyperplasia; other features include the increase of synovial lining cells and infiltration of lymphocytes and macrophages.

From a biochemical perspective, synovial inflammation is sustained by many mediators, in particular Interleukin-1 (IL-1) and tumor necrosis factor (TNF). They induce the synthesis of matrix metalloproteinases (MMPs) and other proteinases but also stimulate the synthesis of cytokines like IL-6, IL-8, IL-15, IL-17, leukemia inhibitory factor, and prostaglandin E2 (PGE_2_) by chondrocytes, synovial cells, and T lymphocytes [49–52].

Synovial neovascularization, driven by an altered expression of pro- and antiangiogenic factors is another important feature of the inflamed synovial membrane; angiogenesis and inflammation are closely related affecting disease progression and pain [53].

## 4. Physiology and Mechanisms of OA Pain

OA related pain may not be similar among individuals since it is a complex process with multifaceted etiologies within and outside the joint and with multidimensional characteristics [54]. The synovium, ligaments, capsule, subchondral bone, and surrounding tissues, with the exception of articular cartilage, are anatomical districts where the presence of pain receptors has been previously demonstrated [54, 55]; thus, they might exert an active role in pain perception in case of OA development.

Patients with OA generally show two distinct types of pain: (1) a dull, aching pain, which becomes more constant over time; (2) intermittent pain varying in intensity. Interestingly, pain experienced by patients changes along with OA stages. In fact, pain progression is activity-induced intermittent in the early stages of OA, while in the intermediate stages, it is frequent and intermittent and progresses to constant pain also interfering with activities of daily living. At the end, advanced OA pain stage is characterized by constant dull/aching pain accompanied by an intense intermittent phase [56]. In addition to idiopathic pain, which is a condition without known cause, pain can be physiologically distinguished in neuropathic or nociceptive. While neuropathic pain is the consequence of nerve damage, nociceptive pain is caused by the activation of nociceptors by noxious stimuli (mechanical, chemical, or thermal) [57]. For a long time OA pain has been considered as nociceptive, but during the last years a growing amount of evidence suggested that neuropathic pain (both peripheral and central sensitization) could be also involved in OA [58, 59]. Moreover, there are data demonstrating that pain perception may also be modulated by the psychological state of the subject [60], as it is a subjective experience [3]. The chronic activation of nociception system in OA is followed by the phenomenon of peripheral sensitization which consists in the decrease of the threshold to elicit a neuronal response. As a consequence, innocuous stimuli such as normal joint movements become painful (allodynia) and the response can be exaggerated (hyperalgesia) [13]. Like peripheral sensitization, central sensitization has been described as a consequence of chronic and continuous nociceptor stimulus leading to hyperexcitability of pain circuits in the central nervous system [61].

Inflammation and mechanopathology are both linked to OA pain but the contribution of each could vary from individual to individual and also from time to time [58].

Moreover, continuous stimulation of pain fibers induces inflammation, which stimulates relative sensory hyperinnervation determining perpetuation of the process of neuroinflammation [62]. Within the joint, there are specific nociceptors that are typically activated by mechanical stimulus and also, as stated above, mechanical stimuli have a role in inducing joint pain due to an increase of intra-articular pressure. If in a normal joint the pressure is between 2 and 10 mm Hg, it can arise up to 20 mm Hg in inflammation or articular lesion. Also cartilage damage may induce hyperpressure of the subchondral bone [55].

### 4.1. The Role of IFP and Synovial Membrane in OA Pain

While it is well known that synovial inflammation is linked to pain, only preliminary data support the association of IFP. Cowan et al. reported that a greater IFP volume in patellofemoral OA was associated with OA pain [63]. Hypointense IFP signal was associated with increased knee cartilage defects, bone marrow lesions, and knee symptoms [64]. Hill et al. evaluated synovial inflammation in 3 different locations (IFP, suprapatellar, and intercondylar regions) showing that changes in IFP were most strongly related to pain change [65]. Since IFP and synovium are intimately related and the clear distinction between these two tissue by MRI being sometimes difficult, a Hoffa-synovitis score was developed in the context of MRI Osteoarthritis Knee Score [66]. Ballegaard et al. observed that both the severity of inflammation in the IFP and MOAKS Hoffa-synovitis were associated with the severity of pain in KOA [67]. From the microscopic point of view, IFP contains both sensory fibers (substance-P positive) and sympathetic fibers (tyrosine hydroxylase positive) [68]. Interestingly, there is a preponderance of sensory over sympathetic innervation in the IFP in anterior knee pain (AKP) patients compared to OA patients, which possibly leads to aggravation and continuation of AKP and local inflammation [68]. However, a detailed description of the IFP changes occurring during OA in the innervation is still missing.

Both ultrasound and MRI have demonstrated the presence of macroscopic synovial inflammation in OA and its association with structural progression and pain [21]. Recently, Sarmonova et al. showed an association between synovitis and knee pain using ultrasound and suggested that effusion but not synovial hypertrophy at baseline predicts knee pain worsening at one year but the prediction is not independent from radiographic OA [69]. Ultrasound synovial changes are related to radiographic severity of OA but the causal relationship has yet to be clearly established [69].

Baker et al. demonstrated the strong relation between synovitis and knee pain severity, an association which is detected clearly with contrast-enhanced MRI. Synovitis, assessed by contrast-enhanced MRI, correlated also with radiographic tibiofemoral OA severity [70]. Moreover, it has to be specified that different patterns of synovitis in KOA has been observed and the pattern, including several patellar sites, which was associated with pain whereas other patterns showed no association, suggesting that pain perception in patients with KOA is a localized response [71].

Interestingly, inflammation, as evidenced by synovitis or effusion, is associated also with pain sensitization in KOA [22]. Therefore, synovial inflammation could drive peripheral and central sensitization in OA, which is consistent with the findings from OA animal models. Thus, targeting synovitis might reduce OA pain [22].

Looking at studies with a mid-long-term follow-up (2-5 years), data are contrasting [21]. There are studies supporting a decrease in pain when synovitis diminishes and a worsening associated with the increase of inflammation. However, this relationship has not been confirmed by other studies [21].

Considering experimental studies, tissue histology and immunohistochemical analysis have confirmed the presence of synovial inflammation in OA [19, 21]. However, studies focused on the relation between synovitis and OA pain are lacking. Certainly, inflammatory mediators of OA can stimulate knee innervating nociceptors but the innervation changes in OA have been poorly investigated. Moreover, we have also to consider that since angiogenesis is intimately related to innervation, it may contribute to synovitis [72]. Healthy synovium contains substance-P and calcitonin gene-related peptide-1 (CGRP-1) perivascular nerves as well as sympathetic nerves containing tyrosine hydroxylase or neuropeptide Y displaying a similar distribution to that of sensory nerves [72]. While it is clear that synovial angiogenesis in OA increases with raising macrophage infiltration and histological grade of inflammation [72], the status of synovial innervation is not clear. Eitner et al. evaluated the inflammatory changes of human OA synovial membrane compared to normal rat and sheep in association with the innervation pattern in the synovial layer and found a massive destruction of the neuronal network related to presence of inflammation in OA [73, 74]. These authors stated that, due to the disappearance of the sensory fibers, it is unlikely that OA pain initiates directly within the synovium [73, 74]. The sensory fibers disappearance is actually supported only by an old study in a mouse model of OA induced by intra-articular injection of collagenase published in 1992 [75]. However, nerves grow more slowly than blood vessels; therefore innervation of new vessels could appear as it was reduced. Hence, synovitis might be associated with reduced immunostaining for markers specific to sensory nerves, particularly when angiogenesis and stromal hyperplasia outstrip the capacity for nerve growth [72]; indeed, we demonstrated that synovial membrane of end-stage OA patients displays an increase of vascularization compared with healthy synovium [19]. In contrast with Eitner et al. [74], Witonski et al. found no differences when evaluating sensory nerve fibers (substance-P positivity) in both synovial membrane and IFP of OA patients compared with patients with posttraumatic knee joint damage [76].

**Figure 3:**
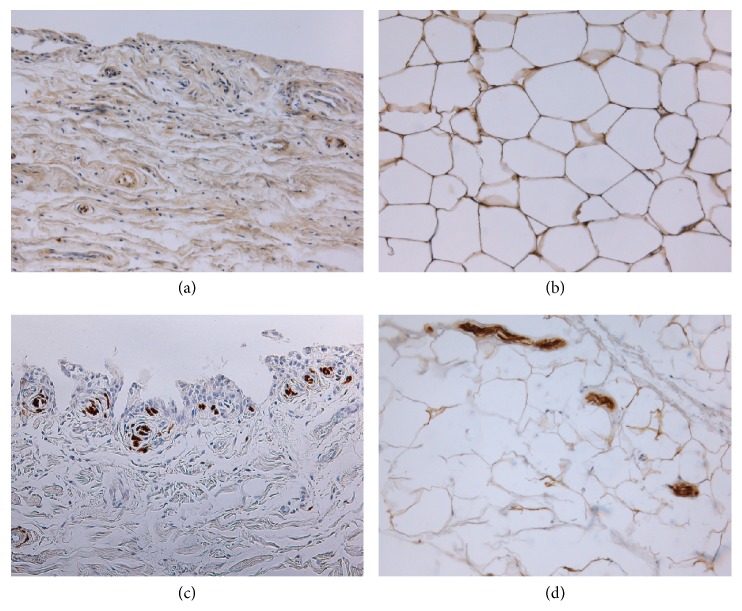
*S-100 immunostaining of infrapatellar fat pad (on the right) and synovial membrane (on the left) in normal subjects and in osteoarthritic patients*. Synovial membrane (a) and infrapatellar fat pad (b) of a healthy subject. Synovial membrane (c) and infrapatellar fat pad (d) of an osteoarthritic patient. In both tissue samples from OA patients immunostaining highlighted an increase of nervous fiber compared to controls. Original magnification 20X.

We have evaluated also the calcium binding protein S-100, a neuronal marker, in both end-stage OA and healthy subjects (unpublished results). Immunohistochemistry allowed observing an increase of nervous fibers in OA compared to controls (Figure 3). Our preliminary data are in accordance with Bohnsack et al. highlighting high number of S-100- and substance-P nerves in the synovium of the anterior knee compartment and in the IFP [77], suggesting that anterior knee pain could originate in compression or chronic inflammation of those tissues. 

## 5. IFP-Synovial Membrane Mediators Involved in OA Pathology and Pain

The molecules involved in OA pain coming from IFP-synovial membrane can be divided into 3 groups: (a) neuropeptides and peptide hormones; (b) growth factors; (c) cytokines.

### 5.1. Neuropeptides and Peptide Hormones

Neuropeptides and peptide hormones are markers of sensory and sympathetic fibers. In particular, two major neuropeptides, substance-P (SP) and calcitonin gene-related peptide (CGRP), are present in sensory nerve fibers (Table 1).

The importance of SP in OA has been recorded as it stimulates the production of inflammatory cytokines (i.e., IL-1*β*, IL-6, and IL-8) from different cell types [78]. Since SP release leads to local inflammation and activation of immune cells, this neuropeptide is actively involved in neurogenic inflammation [79]. In OA, it has been suggested that SP could have a role in neurogenic inflammation but the exact function is still far to be understood [79].

SP-positive nerves have been found in both IFP and synovial tissue [77, 80]. In IFP, SP-positive fibers seem to contribute to the nociceptive function, as they are widely present in IFPs of patients with anterior knee pain and OA [76, 77] and they are probably involved in neurogenic inflammation.

Lotz et al. showed that synovial cells isolated from rheumatoid arthritis and stimulated with SP produced collagenase and PGE_2_ [81]. Walsh et al. showed that SP had a vasodilatatory local function in synovium [82]. In vitro experiments showed that SP could induce rat synoviocytes cell proliferation; thus it has been suggested that this neuropeptide might cause both proliferation and hypertrophy of the synovium within the joint cavity [83]. Interestingly, Okamura et al. showed that synovial mast cells expressed SP and were activated by SP [84]. To this regard, Fusco et al. suggested that mast cells, located within the synovial membrane and along blood vessels as well as near nerve endings, could be directly involved in neuroinflammation, contributing to the shift from acute to chronic pain [85]. Therefore, mast cells could interact both with somatosensory nerve terminals and immune cells amplifying joint inflammation and determining peripheral sensitization of nociceptors and spinal somatosensory neurons [85]. In contrast to SP, sympathetic nerve fibers secrete endogenous opioids and noradrenaline that seem to block the release of SP, other than providing an analgesic effect [68].

CGRP is a vasodilatory neuropeptide present in sensory nerves [86]. Interestingly, it has been demonstrated that CGRP in serum and synovial fluid was related to progressive joint damage in KOA [87]. CGRP positive staining was found in the capillaries of IFP and lining layer of the synovial membrane in OA patients [88, 89]. Its expression has been correlated with ciclo-oxigenase-2 (COX-2) and was higher in IFP than in synovial tissue of OA patients [88]. Additionally, the expression of IFP increased with OA progression suggesting a possible role for CGRP in OA development and pain [88, 90]. Recently, it was shown that CGRP expression in IFP of KOA patients was regulated by the COX-2/mPGES-1/PGE_2_ pathway [91]. Moreover, McMurdo et al. showed that CGRP exerted a vasodilatatory effect in synovial membrane of rat knee joint [92]. Furthermore, it has been reported that CGRP increases proliferation, migration, and tube formation by vascular endothelial cells in vitro as well as in rat knees [93]. However, the link of neurogenic angiogenesis and pain induced by CGRP in human OA patients remains still to be determined. Moreover, CGRP could have a role in the development of synovitis. This is supported by the potentiation of inflammatory mediators by neurologically derived CGRP, as shown in CGRP perfused knee rats [94] and by the inflammation found in knee rat joint injected with capsaicin (a potent CGRP release molecule) [95]. Finally, experimental animal models suggest that CGRP is implicated in OA peripheral sensitization [93].

Neuropeptide Y (NPY) is a small sympathetic neuropeptide widely distributed in the nervous system with its two receptors (Y1 and Y2) located at key pain signaling centres throughout the nervous systems [96]. Positive nerve fibers were located predominantly in the perivascular area of the synovial membrane medial compartment [97]. NPY levels in SF of OA patients were significantly higher compared to controls. Moreover, NPY concentrations correlated with OA severity and pain [98]. Therefore, NPY could be a candidate as pain perception biomarker [96]. However, the contribution of NPY to pain and as potential therapeutic target to counteract pain needs to be further explored.

Vasoactive Intestinal Peptide (VIP) is a vasoactive neuropeptide, member of the secretin/glucagon family, which possesses different biological functions, including immunomodulatory and anti-inflammatory activity, making it interesting in treatments of immune diseases, including arthritis. Juarranz et al. showed VIP mRNA and protein expression in isolated OA synoviocytes [99]. Moreover, VIP positivity was found in lining synoviocytes of OA synovial membrane [99]. Evidence suggests that VIP is shown to prevent chronic cartilage damage and joint remodeling [100] and a downregulation of VIP expression has been found in OA synovial fluid [100]. Interestingly, the injection of a VPAC receptor antagonist VIP (6-28) has been shown to alleviate OA pain, probably counteracting proinflammatory stimuli in a rat model of OA [101, 102]. Thus, it is likely that an imbalance of VIP production in the joint may contribute to the OA pathogenesis and pain. These findings suggest the involvement of VIP in peripheral sensitization of knee joint afferents especially in response to normal joint movements and point out the possible use of this antagonist for the treatment of OA pain [101, 102].

### 5.2. Catecholamines

Joint tissues, such as synovium, are densely innervated by sympathetic nerve fibers. Tyrosine hydroxylase (TH) is the common marker used to identify catecholaminergic nerve fibers [103]. TH positive sympathetic nerve fibers were found in IFP of OA patients [68]. Interestingly, synovial membrane of OA patients has a higher number of sympathetic nerve fibers (TH positive) but less number of TH positive cells (fibroblasts, macrophages, B cells, mast cells, and granulocytes) producing catecholamines than RA patients [104, 105]. Cappellino et al. showed that TH positive cells are present only in inflamed synovial tissue and none in controls. Moreover, these authors demonstrated that TH positive cells produce catecholamines during chronic inflammation with anti-inflammatory effects in vitro as well in vivo opening the possibility of using these cells as a therapeutic target in both OA and RA pathologies [104]. Moreover, since sensory nerve fibers are involved in pain perception and in the secretion of proinflammatory SP, while sympathetic nerve fibers secrete anti-inflammatory catecholamine (in particular norepinephrine and endogenous opioids) inhibiting pain perception, it is likely that a cross-talk with sensory fibers may exist.

### 5.3. Growth Factors

Among growth factors, NGF, VEFG, and TGF-*β*, are discussed (Table 2). NGF is a soluble neuropeptide belonging to neurotrophin family; it is involved in the development of the nervous system (i.e., growth, maintenance proliferation and survival of neurons) but also in pain processing [106, 107] which can be caused by direct and indirect mechanisms [108]. Direct mechanisms involve activation of its high‐affinity receptor (i.e., NGF tyrosine kinase receptor TrkA) located at the surface of sensory neurons [109] and by the low-affinity for receptor p75 [110], followed by activation of different signaling pathways leading to the nociceptive pain [109, 111]. In parallel, another receptor that seems to mediate development of NGF‐induced hyperalgesia is the transient receptor potential channel vanilloid 1 (TRPV1); in fact, the block of TRPV1 attenuates, as demonstrated by Mills et al., thermal and mechanical NGF‐induced hyperalgesia in rats [112]. Interestingly, it functions to modulate OA-associated pain [113] also due to osteochondral angiogenesis [114].

Regarding the effects exerted by NGF on IFP, only a single research paper considered this aspect; according to Munkholm et al. NGF determines mechanical hyperalgesia at the injection site, which do not spread to adjacent areas and is not enhanced by an eventual acid infusion [108].

Concerning the significance of NGF and its receptors in synovial tissue, in 2009, Raychaudhuri et al. used human synovial cells from healthy subjects to consider aspects like their synthesis, release, and functional significance [110]. The existence of a cross-talk between NGF/receptors with fibroblast-like synovial cells was assessed. In particular, upregulation of NGF and TrkA in proinflammatory cytokine-activated synoviocytes, the mitogenic effect of NGF on them, and the increased release of NGF in synovial fluid in case of inflammatory arthritis were verified. Moreover, it was assessed that dysregulated production of NGF determines proliferation of synovial cells influencing the inflammatory and proliferative cascades typical of inflammatory arthritis [110]. More recently, in accordance with Stoppiello et al. who assessed increased synovial NGF in synovitis [115], Montagnoli et al. confirmed a stage-related increase of *β*-NGF and its receptors in the inflammatory process of OA both in serum and synovial fluid [116].

In parallel, Takano et al. suggested that, in the knee of OA patients, NGF production may also be modulated by IL-1b and TNF-a in synovial macrophages and fibroblasts; hence, depleting macrophages to reduce NGF secretion may be considered a future therapeutic approach in OA [113, 117].

VEGF is a mediator of the angiogenic process, essential in the onset and development of joint inflammatory disorders including OA. In fact, it promotes tissue damage and pain both sustaining and increasing the invasion of inflammatory cells and local pain receptors, respectively [118–120]. Moreover, according to genome wide studies, disease severity increases along with VEGF expression [121]. To the best of our knowledge, only our group investigated the IFP/VEGF correlation in OA; hence, in 2017, we assessed higher levels of the growth factor in OA IFP with respect to controls with consequent increase of vessels in the tissue [19]. Considering the synovium, similarly to other areas of the joint (i.e., subchondral bone, articular cartilage) and serum, high levels of VEGF were also encountered in both the synovial membrane (in particular, in synovial lining cells) and synovial fluid of OA patients [120, 122]. Also, VEGF mRNA levels were positively correlated with increased synovial vascularity [19].

According to many research studies in murine models of OA, suppression of VEGF expression may be considered as an interesting potential therapy to treat this disabling disease [121, 123]. Previously, also Heard et al. tried to identify and approach to counteract joint inflammation in OA [124]. Interestingly, the authors considered the IFP as a target; in fact, they tried to mitigate damage descending from IFP injury administering dexamethasone to rabbits used as a model of surgically induced knee injury and inflammation. Unfortunately, intra-articular administration of a glucocorticoid only mitigated the initial inflammation without protecting the joint 9 weeks after surgery as proved by high levels of VEGF mRNA [124].

TGF-*β* is a homodimeric protein, part of a family of over 35 members and in mammalian tissues three peptides are identifiable (i.e., TGF-*β*1, TGF-*β*2, and TGF-*β*3). TGF-*β* exerts its function by binding to receptors located on cell surface and known as TGF-*β* type I (TGFBR1) and TGF-*β* type II (TGFBR2) serine/threonine kinase receptors. To date, in vertebrates the existence of seven TGFBR1 receptors (also termed Activin-receptor-like kinases, ALKs) and five of the TFGBR2 receptors have been recognized [125, 126].

TGF-*β* is responsible for the regulation of many processes from cell proliferation, tissue formation up to repair and inflammation also preserving the differentiated chondrocyte phenotype in normal cartilage. However, its role in health or disease conditions is different, as assessed also in normal or OA joints. This twofold effect, defined by Fang et al. as a “conflicting role”, depends on its concentration and tissue exposure time [125]. In fact, in a normal joint, TGF-*β* presence is identifiable only after loading (which activates it) and for a limited period; while, in case of OA, permanent and high levels of active TGF-*β* occur determining chondrocytes hypertrophy as well as alterations of the subchondral bone [127].

Considering the IFP stromal cells, TGF-*β*1 stimulates the secretion of superficial zone protein (SZP) leading to functional improvement of damaged intra-articular tissues and ameliorating the pathology of joint function in arthritis, thus providing an efficient means to improve joint lubrication [128].

Regarding the relation between TGF-*β* and synovium, high levels of active TGF-*β* were detected in synovial fluid of OA patients in contrast with normal joint. Its activation is due to the acid environment typical of OA which is also responsible for the increase of its levels [129]. In the synovial membrane, TGF-*β* is mainly responsible for fibrosis; hence, as demonstrated by Scharstuhl et al. in mice, inhibition of TGF-*β* activity in experimental OA may inhibit synovial fibrosis [130].

The opposing roles of this protein (i.e., protective towards differentiated chondrocyte in healthy joint and deleterious in an OA joint) make it difficult to identify it as a target for OA therapy [127].

### 5.4. Cytokines and Chemokines

Cytokines are small proteins involved in the interactions between cells and also in the inflammatory response [131]. In particular, considering IFP and synovial membrane, their secretion depends on the immune cell components [132]. Cytokine production, particularly IL‐1*β*, IL‐6, and tumor necrosis factor- (TNF-) *α* have been linked to OA pain and progression [133–136].

Among cytokines, low molecular weight chemokines are recognizable with the role of inducing chemotaxis [131]. Both chemokines and cytokines are produced by joint tissues (synovial membrane, cartilage, meniscus, IFP, and bone), glial cells, and sensory neurons; they are found also in SF exerting an important role in OA disease [17, 137–139]. Moreover, there is evidence that some of them are involved not only in inflammation but also in the process of neuropathic pain [131]. Similarly, also chemokines seem to be involved in the pathogenesis of pain by modulating neuronal activity in the peripheral and central nervous systems [131].

Interleukins are a large family of cytokines whose expression was first described in monocytes and neutrophils. According to their structure and function, four major groups can be recognized including the superfamily of IL-1 [140]. IL-1 comprises 11 members that can be divided into 7 proinflammatory agonists (IL-1*α*, IL-1*β*, IL-18, IL-33, IL-36*α*, IL-36*β*, and IL-36*γ*) and 4 defined antagonists (IL-1R antagonist (IL-1Ra), IL-36Ra, IL-37, and IL-38) showing anti-inflammatory activities [141].

Among these, IL-1*β* has to be considered in case of OA as it is known to have a specialized role as proinflammatory cytokine [142], which promotes cartilage degeneration by enhancing the production of inducible nitric oxide synthase (iNOS), PGE_2_, and COX-2 in human chondrocytes [143]. Moreover, it is responsible for the increased synthesis of NGF, IL-6, and IL-8 and various other cytokines [111]. There is evidence that IL-1*β* has rapid and delayed effects on dorsal root ganglia neurons, from in vitro studies, contributing to hyperexcitability of pain sensory neurons [144].

Considering the synovial membrane, IL‐1*β*‐producing macrophages regulate calcitonin receptor‐like receptor (CLR) expression in synovial cells and may contribute to the pain in OA patients; in fact, calcitonin gene‐related peptide (CGRP) seems to be involved in both pain transmission and neurogenic inflammation [133].

Concerning a possible therapeutic approach, IL-1*β* inhibitors have been tested in animal models and including clinical trials with contrasting results [144]. In particular, the most promising IL 1*β*-receptor antagonist, anakinra was tested in a multicenter, double-blind, placebo-controlled study enrolling patients with KOA without improvements in OA symptoms compared with placebo [145]. The same antagonist was administrated in knee patients suffering of anterior cruciate ligament tear, which is one of the risks factors for OA development; an improvement in clinical outcome was observed compared to patients treated with placebo [146]. Thus, this study increases confidence in IL-1*β* as a promising target for symptoms and potential disease modification following severe joint injury. Moreover, it suggests that an early inhibition of IL-1*β* action could be important to decrease the risk of developing OA [144, 146].

IL-6, in addition to other cytokines belonging to the IL-6 family of protein (i.e., oncostatin M and adiponectin), is considered one of the most represented cytokines involved in OA inflammation [147]. It is involved in cartilage degradation, but also in hypersensitivity, hyperalgesia in the joints, and pain [148]. Regarding its mechanism of action, IL-6 interacts with a membrane-bound IL-6 receptor (mIL-6R*α*) or with a soluble IL-6 receptor (sIL-6R) termed the “*trans*-signaling” pathway [147]. In several studies its presence was observed in the IFP of OA patients suggesting that the local production of IL-6 and sIL-6R by the IFP tissue of obese OA patients may contribute to paracrine inflammation and progressive cartilage damage [19, 149, 150]. More recently, Klein-Wieringa et al. proved that its secretion is due to CD4+ T cells of the IFP [132].

There are many studies illustrating the role of IL-6 in pain, and it has been reported that the administration of IL-6 determines mechanical allodynia or thermal hyperalgesia [151]. Moreover, it has been shown that there is a link between IL-6 and nociceptive plasticity as well as between IL-6 and nociceptor sensitization and also with central sensitization [151]. Therefore, targeting directly IL-6 receptors could represent a response to counteract pain. Interestingly, tocilizumab, a recombinant humanized IL-6 monoclonal antibody, has been successfully used to attenuate pain and neural activity in a rat induced-OA model [152].

TNF-*α* is an inflammatory cytokine which plays a well-established role in several pain models [131] as it is involved in inflammatory and neuropathic hyperalgesia, and it can induce long-term change expression in sensory neurons [131, 144]. The high expression of the p55 TNF-*α* receptor by articular chondrocytes in case of OA suggests the important role of TNF-*α* in the disease, as it is responsible for OA cartilage susceptibility to its degradative stimuli.

Interestingly, the important role of TNF-*α* in OA may emerge from the fact that human articular chondrocytes from OA cartilage expressed a significantly higher number of the p55 TNF-*α* receptor which could make OA cartilage particularly susceptible to TNF-*α* degradative stimuli [153]. In addition, OA cartilage produces more TNF-*α* and TNF-*α* convertase enzyme (TACE) mRNA than normal cartilage [153]. TNF-*α* was detectable in SF and, importantly, it was correlated with pain [154].

Interestingly, the use of etanercept, a TNF-*α* inhibitor, or the monoclonal human anti-TNF-*α* antibody, infliximab, was able to reduce pain associated with the antigen-induced model of inflammatory arthritis in rats, but without inhibiting joint damage [144]. In humans, serum levels of TNF-*α* were similar between OA patients and healthy controls [148]. Nonetheless, small clinical trials have been performed to test TNF-*α* inhibitors or antibody in OA patients with contrasting results [155]. Short-term treatment with adalimumab for 12 weeks in patients with inflammatory OA resulted in clinical benefit in the majority of patients using the OARSI/OMERACT responder index [155]. However, there are two studies showing that it was not effective in patients with hand OA [156]. Interestingly, etanercept treatment in patients with erosive OA of the hands provided promising results regarding the ability of this drug to improve pain and modify structural damage [156].

IL-8 is an inflammatory chemokine that induces chemotaxis and degranulation of neutrophils, as well as stimulation of the release of inflammatory cytokines, including IL-6 and IL-1*β*, contributing to the modulation of the inflammatory response [157]. IL-8 concentration in SF of OA patients has been found to be increased compared to the SF levels, but not serum levels, in posttraumatic patients; no correlation was found with OA severity grades and pain [157].

Also, chemokine (C-C motif) ligand 5 (CCL5/RANTES) is involved in monocytes, eosinophils, and T cells attraction, and it activates both eosinophils and basophils to release the granule content [157]. No differences were found in CCL5/RANTES serum and SF levels compared to both posttraumatic and healthy controls and no correlations were identified with WOMAC index and Altman scale [157]. Therefore, both IL-8 and CCL5/RANTES do not seem to be involved in OA pain.

## 6. Conclusion

OA is a complex painful, multifaced, and disabling disease. Today, the only effective treatment for end-stage KOA patients is the total joint replacement. Since the life expectancy of population and obesity incidence are rising also the prevalence of OA is expected to grow in the next years. Therefore, understanding OA etiopathogenesis and pain mechanisms is a priority in order to identify new therapeutic targets to counteract and manage OA pain.

Inflammation of IFP and synovial membrane within the knee may have a central role in OA pain and may drive peripheral and central sensitization in KOA. Since sensitization is associated with pain severity in KOA and may potentially contribute to the transition from acute to chronic, persistent pain in KOA, preventing sensitization would be a potentially effective and novel means of preventing worsening of pain in knee OA. Early targeting of inflammation in knee OA may therefore be a reasonable strategy to prevent the sensitization and thereby reduce pain severity.

We still need to understand more about individual symptoms and their relationship to particular structural pathologies. Importantly, pain is a complex experience in which changes may be attributed to several peripheral nociceptive factors other than inflammation, as well as central factors.

Evidence suggests that both IFP and synovial membrane contribute to OA pain but today the information about the detailed role of each tissue, as well as the innervation and molecules involved, has yet to be fully investigated and resolved.

## Figures and Tables

**Table 1:** Main neuropeptides and neurotransmitters involved in OA pathology and pain.

		IFP			Synovial Membrane		
Molecule	Generic Function	Localization	Findings and Function		Localization	Findings and Function	
		Humans	IFP adipocytes/animal models	OA pathology and pain in humans	Humans	synoviocytes/animal models	OA pathology and pain in humans
*Neuropeptides/* *peptides hormones *							

Substance P	(i) Act directly on vascular endothelial and smooth muscle cell (ii) Increases capillary permeability leading to plasma extravasation and edema (iii) Inflammation by increase of cytokines expression (iv) Activation of immune cells (v) Involvement in neurogenic inflammation	Sensory nerve fibers equally distributed and frequently associated with blood vessels Bohnsack et al. 2005, Witonski et al 2005 [76, 77].	NA	(i) Nociceptive function (ii) Neurogenic Inflammation? Bohnsack et al. 2005, Witonski et al 2005 [76, 77]	Free nerve endings and sensory fibers widely distributed Saito et al. 2000, Bohnsack et al. 2005, Witonski et al 2005 [76, 77, 80]	(i) Stimulate release of proinflammatory cytokines (Dirmeier et al 2008) [78] (ii) Induce synoviocyte proliferation and synovial hyperthrophy (Matayoshi et al 2005) [83] (iii) Activate synovial mast cells (Okamura et al 2017) [84] (iv) Vasodilatation (Walsh et al. 1992) [82]	(i) Neurogenic inflammation ? (Saito 2003, Fusco et al 2017) [85, 97]

CGRP	(i) Vasodilation (ii) Nociceptive function	Capillaries (Aikawa et al 2017) [88]	NA	(i) Expression increases as pain increases (Aikawa et al 2017) [88] (ii) Expression levels of were positively correlated with COX-2 (Aikawa et al 2017) [88] (iii) Expression levels are regulated by the COX-2/mPGES-1/PGE2 pathway (Aikawa et al 2018) [91]	Synovial fibroblasts of lining layer (Aikawa et al 2017; Takano et al 2017) [88, 90]	(i) Vasodilatation (McMurdo et al 1997) [92] (ii) Angiogenesis (Walsh et al 2015) [93] (iii) Inflammation (Cruwys et al 1992; Mapp et al 1996) [94, 95] (iv) Peripheral sensitization (Walsh et al 2015 ) [93]	(i) expression levels is lower compared to IFP levels (Aikawa et al 2017) [88] (ii) expression is regulated by the COX-2/PGE2 pathway and that elevation of synovial CGRP levels may contribute to OA pain (Takano et al 2017) [90]

NPY	(i) Food intake (ii) Cardiovascular performance (iii) Neuropathic pain (iv) inflammation	NA	NA	NA	Positive sympathetic nerve fibers were located predominantly in the perivascular area of medial compartment (Saito 2003) [97]	NA	Neurogenic inflammation ?

VIP	(i) immunomodulatory (ii) anti-inflammatory activity	NA	NA	NA	Lining synoviocytes (Juarranz et al 2008) [99]	(i) Expression is evident in cultured OA synoviocytes (Juarranz et al 2008) [99] (ii) Peripheral sensitization in rat models (McDougall 2006, Schuelert et al 2006) [101, 102]	Peripheral sensitization?

CATHECOLAMINES	(i) Neuromodulation in central nervous system (ii) Hormones in blood circulation (iii) Pain perception inhibition (iv) Anti-inflammatory	TH positive sympathetic nerve fibers (Lehner et al 2007) [68]		Anti-inflammatory ? Inhibition of pain perception?	Sympathetic nerve fibers and TH positive cells (fibroblasts, mast cells, granulocytes, macrophages, B cells) only in OA not in controls (Capellino et al 2010) [104]	anti-inflammatory effects in vitro and in vivo by inhibiting pro-inflammatory cytokines (Capellino et al 2010) [104]	(i) Anti-inflammatory? (ii) Inhibition of pain perception?

? = suggested function.

**Table 2:** Main growth factors involved in OA pathology and pain.

Category/Molecule	General description	References	IFP	References	Synovial membrane	References
*Growth factors*						

NGF	(i) soluble neuropeptide of neurotrophin family (ii) it is involved in the development of the nervous system (iii) it is involved in pain processing by direct or indirect mechanisms (iv) Responsible of OA-associated pain and osteochondral angiogenesis	Lewin and Mendell, 1993; Walsh et al., 2010; Takano et al., 2017; Shang et al., 2017; Berenbaum 2018 [106, 109, 111, 114]	(i) NGF determines mechanical hyperalgesia at the injection site, but it do not spread to adjacent areas and is not enhanced by acid infusion	Munkholm and Arendt-Nielsen 2016 [108]	(i) Upregulation of NGF and TrkA in proinflammatory cytokine-activated synoviocytes, (ii) the mitogenic effect of NGF on synoviocytes (iii) increased release of NGF in synovial fluid in case of inflammatory arthritis; (iv) increased synovial NGF in synovitis (v) a stage-related increase of *β*-NGF and its receptors in OA (vi) IL-1b and TNF-a may modulate NGF production in synovial macrophages and fibroblasts	Raychaudhuri et Raychaudhuri 2009; Stoppiello et al., 2014; Montagnoli et al., 2017; Takano et al., 2016, 2017 [110, 113, 115–117]

VEGF	(i) mediator of the angiogenic process; (ii) essential in joint inflammatory disorders; (iii) it promotes tissue damage and pain	MacDonald et al., 2018; Takano et al.,2018; Deng et al., 2018 [3, 118, 119]	(i) higher in OA IFP versus controls; (ii) increase of vessels in the tissue.	Favero et al., 2017 [19]	(i) high levels in the synovial membrane and fluid of OA patients (ii) VEGF mRNA levels were positively correlated with increased synovial vascularity	Takano et al., 2018; Favero et al., 2017; Ding et al., 2017 [19, 120, 122]

TGF-beta	(i) homodimeric protein (ii) in mammalian tissues three peptides are identifiable (i.e. TGF-*β*1, TGF-*β*2, and TGF-*β*3). (iii) It exerts its function by binding to TGF-*β* type I (TGFBR1) and TGF-*β* type II (TGFBR2) serine/threonine kinase receptors. (iv) It regulates many cellular process (v) its role in health or disease conditions is different depends on its concentration and tissue exposure time	Yingling et al., 1995; Fang et al.,2016; van der Kraan 2018 [125–127]	(i) TGF-*β*1 stimulates the secretion of SZP	Lee et al., 2008 [68]	(i) high levels of active TGF-*β* were detected in synovial fluid of OA patients (ii) mainly responsible for fibrosis	Zielinski et al., 2000 Scharstuhl et al. 2003 [129, 130]
